# The tidal volume challenge improves the reliability of dynamic preload indices during robot-assisted laparoscopic surgery in the Trendelenburg position with lung-protective ventilation

**DOI:** 10.1186/s12871-019-0807-6

**Published:** 2019-08-07

**Authors:** Joo-Hyun Jun, Rack Kyung Chung, Hee Jung Baik, Mi Hwa Chung, Joon-Sang Hyeon, Young-Goo Lee, Sung-Ho Park

**Affiliations:** 1 Department of Anesthesiology and Pain Medicine, Kangnam Sacred Heart Hospital, Hallym University, College of Medicine, Seoul, South Korea; 2 Department of Anesthesiology and Pain Medicine, Ewha Womans University, College of Medicine, Anyangcheon-ro, Yangcheon-gu, Seoul, 1071 South Korea; 3 Department of Urology, Kangnam Sacred Heart Hospital, Hallym University, College of Medicine, Seoul, South Korea; 4 Department of Obstetrics and Gynecology, Kangnam Sacred Heart Hospital, Hallym University, College of Medicine, Seoul, South Korea

**Keywords:** Pulse pressure variation, Stroke volume variation, Fluid responsiveness, Tidal volume challenge, Pneumoperitoneum, Trendelenburg position

## Abstract

**Background:**

The reliability of pulse pressure variation (PPV) and stroke volume variation (SVV) is controversial under pneumoperitoneum. In addition, the usefulness of these indices is being called into question with the increasing adoption of lung-protective ventilation using low tidal volume (V_T_) in surgical patients. A recent study indicated that changes in PPV or SVV obtained by transiently increasing V_T_ (V_T_ challenge) accurately predicted fluid responsiveness even in critically ill patients receiving low V_T_. We evaluated whether the changes in PPV and SVV induced by a V_T_ challenge predicted fluid responsiveness during pneumoperitoneum.

**Methods:**

We performed an interventional prospective study in patients undergoing robot-assisted laparoscopic surgery in the Trendelenburg position under lung-protective ventilation. PPV, SVV, and the stroke volume index (SVI) were measured at a V_T_ of 6 mL/kg and 3 min after increasing the V_T_ to 8 mL/kg. The V_T_ was reduced to 6 mL/kg, and measurements were performed before and 5 min after volume expansion (infusing 6% hydroxyethyl starch 6 ml/kg over 10 min). Fluid responsiveness was defined as ≥15% increase in the SVI.

**Results:**

Twenty-four of the 38 patients enrolled in the study were responders. In the receiver operating characteristic curve analysis, an increase in PPV > 1% after the V_T_ challenge showed excellent predictive capability for fluid responsiveness, with an area under the curve (AUC) of 0.95 [95% confidence interval (CI), 0.83–0.99, *P* < 0.0001; sensitivity 92%, specificity 86%]. An increase in SVV > 2% after the V_T_ challenge predicted fluid responsiveness, but showed only fair predictive capability, with an AUC of 0.76 (95% CI, 0.60–0.89, *P* < 0.0006; sensitivity 46%, specificity 100%). The augmented values of PPV and SVV following V_T_ challenge also showed the improved predictability of fluid responsiveness compared to PPV and SVV values (as measured by V_T_) of 6 ml/kg.

**Conclusions:**

The change in PPV following the V_T_ challenge has excellent reliability in predicting fluid responsiveness in our surgical population. The change in SVV and augmented values of PPV and SVV following this test are also reliable.

**Trial registration:**

This trial was registered with Clinicaltrials.gov, NCT03467711, 10th March 2018.

## Background

Robot-assisted laparoscopic surgery is increasingly performed due to its many advantages over open surgery, including minimal tissue trauma, fewer surgical complications, and earlier postoperative recovery [1]. However, pneumoperitoneum is essential for adequate exposure in robot-assisted laparoscopic surgery, which has been associated with increased morbidity such as reduced renal blood flow (RBF) and post-operative renal dysfunction [2, 3]. As the level of hydration required to maintain RBF under pneumoperitoneum depends on the baseline volume status [4], an adequate assessment of intravascular volume and optimal fluid management are important [5]. However, predicting the fluid responsiveness with clinical signs, such as blood pressure, or urine output or cardiac filling pressure is more difficult during pneumoperitoneum [6, 7].

Dynamic preload indices such as pulse pressure variation (PPV) and stroke volume variation (SVV), are generally accepted as accurate indicators of fluid responsiveness during surgery and in the intensive care unit (ICU) in patients ventilated with tidal volume (V_T_) of at least 8 ml/kg or more of predicted body weight (PBW) [8–10]. As dynamic preload indices are generated by cyclic transmission of airway pressure to the pleural and pericardial spaces under positive ventilation, their reliability can be affected by extra-thoracic modification in compliance such as pneumoperitoneum. Several studies have highlighted the effect of intra-abdominal pressure (IAP) on the accuracy and cut-off values of these indices [11–15]. In addition, as the application of lung-protective ventilation using low V_T_ with positive end expiratory pressure (PEEP) is gradually increasing in surgical patients [16–18], the usefulness of these indices during robot-assisted laparoscopic surgery has been questioned.

Recently, Myatra et al. developed a functional test called the “V_T_ challenge”, which includes transiently increasing V_T_ from 6 ml/kg to 8 ml/kg PBW and observing the changes in PPV and SVV [19]. They demonstrated that the absolute changes of PPV and SVV values induced by V_T_ challenge, as well as their augmented values, predicted fluid responsiveness with high sensitivity and specificity, even in critically ill patients receiving low V_T_ [19]. This test also validated in predicting fluid responsiveness in surgical patients receiving low V_T_ [20].

Therefore, the aim of the current study was to investigate the ability of absolute changes in PPV and SVV values induced by V_T_ challenge to predict fluid responsiveness in patients undergoing robot-assisted laparoscopic surgery in the Trendelenburg position under lung-protective ventilation. We also assessed the whether augmented values of PPV and SVV following this test are also reliable to predict fluid responsiveness.

## Methods

### Study design and patient population

This interventional prospective study was approved by the institutional review board of Hallym University Kangnam Sacred Heart Hospital (approval number: 2017–09-003). From March to June 2018, adult patients undergoing robot-assisted laparoscopic surgery with pneumoperitoneum in the Trendelenburg position were enrolled after obtaining their written informed consent. The trial was registered prior to patient enrollment at ClinicalTrials.gov (NCT03467711). Exclusion criteria were body mass index (BMI) > 30 or < 15 kg/m^2^, preoperative arrhythmia, moderate to severe valvular heart disease, preoperative left ventricular ejection fraction < 40%, right ventricular dysfunction, intracardiac shunt, 1-s forced expiratory volume < 60% of predicted value, moderate to severe renal or liver disease, new-onset arrhythmia after anesthesia induction, and contraindications for oesophageal Doppler monitor (ODM) probe insertion (i.e., oesophageal stent, carcinoma of the esophagus or pharynx, previous oesophageal surgery, oesophageal stricture, oesophageal varices, pharyngeal pouch, and severe coagulopathy). During surgery, all patients were placed in the 25° Trendelenburg position, and pneumoperitoneum was achieved by continuous carbon dioxide insufflation maintaining an IAP of 15 mmHg.

### Anesthetic technique

After the patients arrived at the operating room, pulse oximetry, three-lead electrocardiography (ECG), and non-invasive arterial pressure monitoring were applied. Anesthesia was induced with propofol (1.5–2.5 mg/kg) and remifentanil (0.05–0.15 μg/kg/min), and tracheal intubation was facilitated with rocuronium (0.8 mg/kg). The patient’s lungs were mechanically ventilated with a mixture of oxygen in air, with an inspired oxygen fraction of 0.5 using the volume-controlled mode. V_T_ was adjusted to 6 ml/kg PBW (determined as x + 0.91[height (in cm) − 152.4], where x = 50 for males and x = 45.5 for females) [21]. The PEEP of 5 cm H_2_O was applied without inspiratory pause. Respiratory rate (RR) was adjusted to maintain an end-tidal carbon dioxide tension between 35 and 40 mmHg. The inspiratory to expiratory ratio was set to 1:2. Peak inspiratory airway pressure (PIP) and compliance of the respiratory system (Crs) were recorded from the anesthesia machine (Datex-Ohmeda Avance CS^2^ Anesthesia Machine; GE Healthcare, Helsinki, Finland). Anesthesia was maintained with continuous infusion of remifentanil (0.02–0.2 μg/kg/min) and sevoflurane (1.5–2.5 vol%) to maintain a bispectral index between 40 and 60.

### Hemodynamic monitoring

After induction of anesthesia, a radial arterial catheter and ODM probe (CardioQ; Deltex Medical, Chichester, UK) were inserted. Both were connected to the CardioQ-ODM+ (Deltex Medical Ltd.) monitor.

The ODM probe was positioned to obtain the optimum signal for descending aorta blood velocity. Stroke volume index (SVI), and peak velocity (PV) were measured continuously and displayed, and their mean values were calculated over 10 s.

After zeroing the arterial transducer, a flush test was performed to ensure that the arterial pressure measurement system was critically damped. The arterial pulse pressure wave was simultaneously monitored through the patient monitor (CARESCAPE Monitor B850; GE Healthcare) and CardioQ-ODM+ monitor using a serial cable. The patient monitor displayed the automatically calculated PPV in real time using the algorithms described previously [22].

$$ \mathrm{PPV}\ \left(\%\right)=\left[\left({\mathrm{PP}}_{\mathrm{max}}-{\mathrm{PP}}_{\mathrm{min}}\right)/{\mathrm{PP}}_{\mathrm{mean}}\right]\times 100 $$

where PP_max_ and PP_min_ represent the maximum and minimum arterial pulse pressure (PP), and PP_mean_ is the mean arterial PP.

The CardioQ-ODM+ monitor combines ODM with pulse pressure wave analysis to measure SVI. It uses ODM-derived SVI for initial and periodic calibrations, and then continuously calculates pulse pressure wave analysis-derived SVI using the Liljestrand–Zander formula [23]. By continuous beat detection and analysis, the SV, SVI, and SVV were displayed continuously in a separate pressure-based data window as a column of values. SVV were obtained as described previously, regardless of the respiratory cycle [24].

$$ \mathrm{SVV}\ \left(\%\right)=\left[\left({\mathrm{SV}}_{\mathrm{max}}-{\mathrm{SV}}_{\mathrm{min}}\right)/{\mathrm{SV}}_{\mathrm{mean}}\right]\times 100 $$

where SV_min_ and SV_max_ are the minimum and maximum SV values over one respiratory cycle, respectively.

All values were averages of at least three consecutive measurements acquired over 30 s. An independent investigator who was trained in maneuvering the ODM probe but was not involved in the present study assessed ODM and all other variables during the study. ODM is routinely used to monitor surgical patients in our center and shows good inter-observer reliability [14].

### Study protocol

**Fig. 1:**
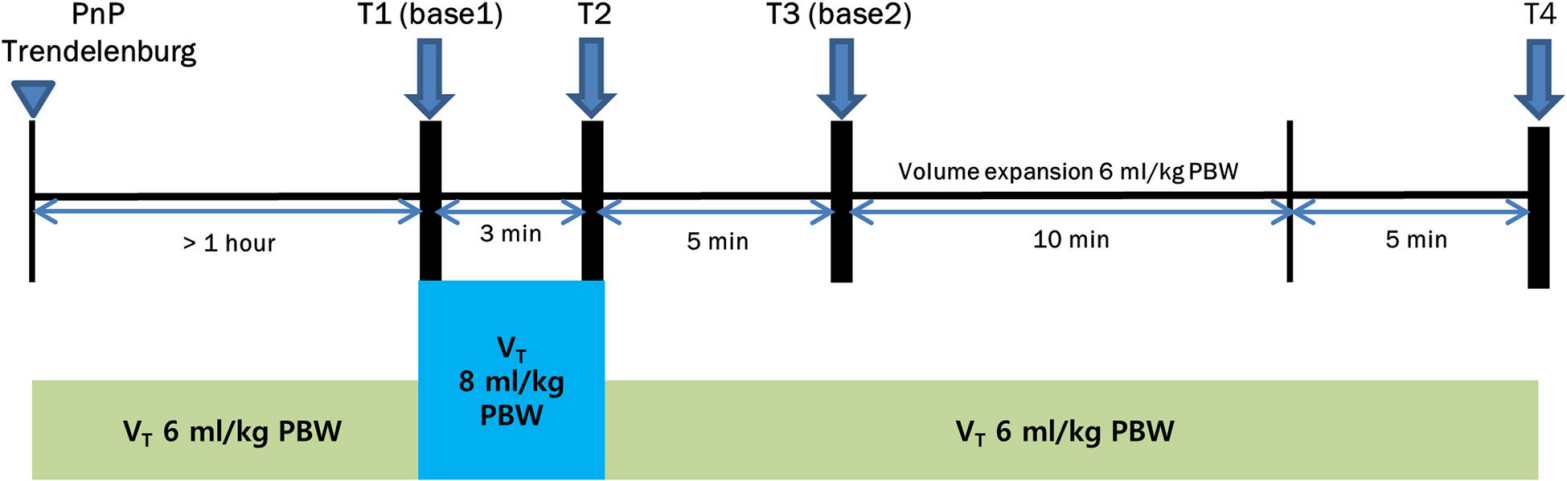
Study protocol. Arrows indicate time points at which measurements were made. PnP, pneumoperitoneum; V_T_, tidal volume; PBW, predicted body weight

Figure 1 shows a schematic representation of the protocol, which was initiated at least 1 h after increasing IAP to 15 mmHg, and after stabilization of hemodynamic parameters, defined as changes in mean arterial pressure (MAP) < 10% during 5 min. In addition, to minimize acute changes in IAP and sympathetic tone due to ongoing surgery [25, 26], which could confound the effects of fluid challenge, the study protocol was performed with little or no surgical stimulation (absence of cautery and instrumentation of intra-abdominal structures).

We first measured the hemodynamic response to V_T_ challenge, and then performed volume expansion (VE) to assess the subsequent changes in SVI. The first set of measurements, including (HR), MAP, SVI, PV, PIP, *C*_rs_, PPV with 6 ml/kg PBW V_T_ ventilation (PPV_6_), and SVV with 6 ml/kg PBW V_T_ ventilation (SVV_6_), were recorded at baseline (T1, base 1). After the baseline measurement, V_T_ was increased from 6 to 8 ml/kg PBW for 3 min without changing the RR. During the last minute of high V_T_ ventilation, measurements of the above-mentioned hemodynamic and respiratory variables, including PPV with 8 ml/kg PBW V_T_ ventilation (PPV_8_) and SVV with 8 ml/kg PBW V_T_ ventilation (SVV_8_), were again recorded (T2). The changes in PPV and SVV values induced by V_T_ challenge (ΔPPV_6–8_ and ΔSVV_6–8_) were calculated as follows:

$$ {\Delta \mathrm{PPV}}_{6-8}={\mathrm{PPV}}_8-{\mathrm{PPV}}_{6,} $$

$$ {\Delta \mathrm{SVV}}_{6-8}={\mathrm{SVV}}_8-{\mathrm{SVV}}_{6.} $$

The percentage change in PPV and SVV values induced by V_T_ challenge (%ΔPPV_6–8_ and %ΔSVV_6–8_) also calculated.

After the V_T_ was returned to 6 ml/kg PBW and all of the hemodynamic variables had returned to baseline values (variations < 10%), VE was performed for 10 min using an infusion of 6% hydroxyethyl starch (HES 130/0.4, Volulyte; Fresenius Kabi, Stans, Switzerland) 6 ml/kg PBW. Two sets of measurements (HR, MAP, SVI, PV, PIP, Crs, PPV, and SVV) were performed before (T3, base 2) and 5 min after VE (T4) [27, 28]. Percentage differences in ODM-derived SVIs before and after VE were used as principal indicators of fluid responsiveness. Patients were classified as responders to VE if they showed an increase in SVI ≥ 15% and as non-responders if they showed an increase < 15% [29, 30]. The changes in SVV and PPV values after VE (ΔPPV_VE_ and ΔSVV_VE_) were calculated as follows:

$$ {\Delta \mathrm{PPV}}_{\mathrm{VE}}=\mathrm{PPV}\ \left(\mathrm{T}4\right)-\mathrm{PPV}\ \left(\mathrm{base}\ 2,\mathrm{T}3\right), $$

$$ {\Delta \mathrm{SVV}}_{\mathrm{VE}}=\mathrm{SVV}\ \left(\mathrm{T}4\right)-\mathrm{SVV}\ \left(\mathrm{base}\ 2,\mathrm{T}3\right). $$

### Statistical analysis

MedCalc for Windows (ver. 15.6.1; MedCalc Software, Ostend, Belgium) was used to calculate sample size. The sample size was determined using the difference between the area under the curve (AUC) of 0.75 (alternative hypothesis that PPV_8_ can predict fluid responsiveness after VE) and 0.5 (null hypothesis). At least 38 patients were required to detect an AUC difference of 0.25 with a type I error of 0.05 and a desired power of 0.80, assuming equal numbers of responders and non-responders. With the expectation of a 10% dropout rate, 42 patients were enrolled in the study.

The normality of the continuous data was tested with the Shapiro–Wilk test. Data are presented as the mean (SD), median [interquartile range (IQR)], or number of patients (%).Student’s *t*-test or the Mann–Whitney U test for continuous variables, and the chi-square test for categorical data, were used to compare patient characteristics between responders and non-responders. The hemodynamic parameters were compared between responders and non-responders using the Mann–Whitney U-test or *t*-test, as appropriate. The effects of the temporary increase in V_T_ from 6 to 8 ml/kg and VE on hemodynamic parameters were assessed using the paired *t*-test or the Wilcoxon signed-rank sum test after the normality test. A Bonferroni-adjusted *P*-value (normal *P*-value multiplied by the number of outcomes being tested) was used to control for multiple comparisons.

The relations between percentage changes in SVI after VE and hemodynamic parameters before VE (PPV_6_, PPV_8_, ΔPPV_6–8_, SVV_6_, SVV_8_, and ΔSVV_6–8_) were assessed using Spearman’s rank correlation. The relationship between the percentage changes in SVI after and the changes in PPV and SVV after VE (ΔPPV_VE_ and ΔSVV_VE_) were also assessed using Spearman’s rank correlation analysis. The intraclass correlation between the SVI, PPV, and SVV measurements at the two baseline steps (T1 and T3) was measured using random-effects models [31].

To test the abilities of dynamic preload indices to predict fluid responsiveness, the AUCs of receiver operating characteristic (ROC) curves were calculated and compared using the DeLong method. Briefly, the general interpretations of a test according to the value of the AUC of the ROC were as follows: AUC = 0.5, no better than chance, a useless test with no prediction possible; AUC = 0.6–0.69, a test with a poor predictive capability; AUC = 0.7–0.79, a fair test; AUC = 0.8–0.89, a test with good predictive capability; AUC = 0.9–0.99, an excellent test; AUC = 1.0, a perfect test with the best possible prediction. An optimal threshold value was determined for each variable to maximize the Youden index (sensitivity + specificity – 1). Considering the possibility of an overlap between responders and non-responders, we determined a grey zone for dynamic preload indices, considering a low cut-off value including 90% of negative fluid challenge responses, and a high cut-off value predicting positive fluid challenge in 90% of cases [32].

Statistical analyses were performed using MedCalc (ver. 15.6.1) and SPSS software (ver. 24.0; IBM Corp., Armonk, NY, USA) and R package (version 3.4.2; https://www.r-project.org/). In all analyses, *P* < 0.05 was taken to indicate statistical significance.

## Results

### Patient characteristics

**Fig. 2:**
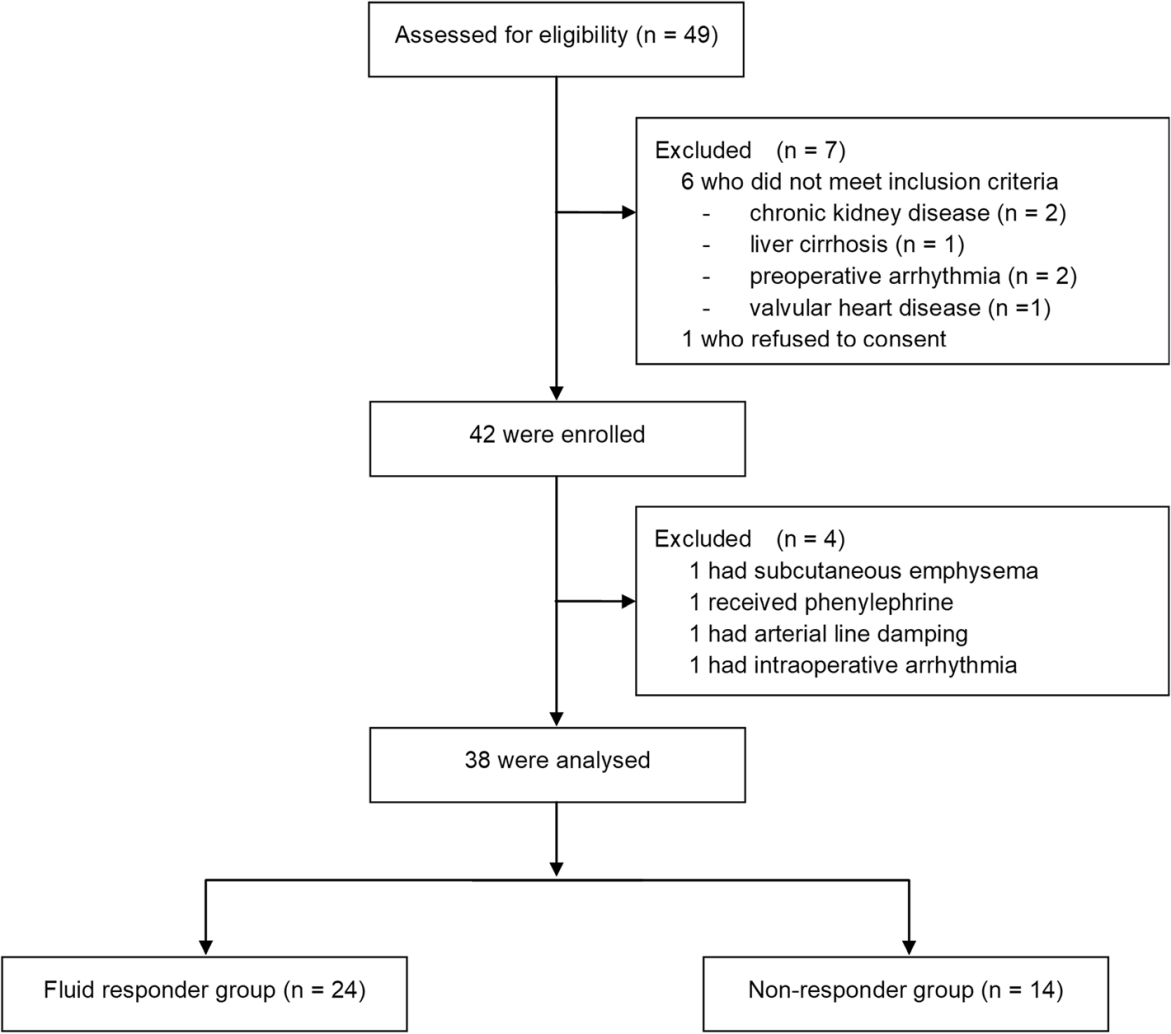
Study diagram

Of the 49 patients included in the initial screen, 42 fulfilled the inclusion criteria and were enrolled in the study. Four patients were excluded; one developed intraoperative subcutaneous emphysema and required a ventilator mode change, one had severe hypotension during VE and required vasopressor support, one developed paroxysmal atrial fibrillation during surgery, and the remaining patient’s arterial pressure measurement system was critically damped. Among the 38 patients included in the final analysis, 24 patients (63%) were responders and 14 (37%) were non-responders (Fig. 2). There were no significant differences in age, PBW, or BMI between responders and non-responders, whereas the surgery type and sex distribution differed between the two groups (Table 1). The intraclass correlation between the SVI, PPV, and SVV measurements at the two baseline steps (T1 and T3) were 0.98 [95% confidence interval (CI), 0.96–0.99], 0.96 (95% CI, 0.92–0.98), and 0.81 (95% CI, 0.64–0.90), respectively.

**Table 1:** Patient Characteristics

	Overall (*N* = 38)	Responders (*n* = 24)	Non-responders (*n* = 14)	*P*-value
Age, years (range)	49.5 (45–62.3)	56.5 (45.3–62.8)	47 (41–52.5)	0.058
Sex (male/female)	17/21	14/10	3/11	0.043
Height, cm	162 ± 7.0	163.5 ± 7.2	161.5 ± 6.9	0.406
Predicted body weight, kg	57 ± 7.8	58.26 ± 8.0	54.8 ± 7.2	0.717
Body mass index, kg /m^2^	24.4 ± 2.5	24.5 ± 2.2	24.2 ± 2.9	0.664
Hypertension	9 (29)	6 (28.6)	3 (30)	1.000
Surgery type, n (%)				0.102
Radical prostatectomy	18 (47.4)	15 (62.5)	3 (21.4)	
Hysterectomy	14 (36.8)	6 (25)	8 (57.1)	
Myomectomy	6 (15.8)	3 (12.5)	3 (21.4)	

Values are mean ± SD, median (IQR) or number (%)

### Effects of V_T_ challenge and VE on hemodynamic and respiratory variables

At baseline, with 6 ml/kg PBW V_T_ ventilation, no significant differences were found in any hemodynamic variables, including PPV_6_ and SVV_6_, between responders and non-responders. After increasing V_T_ to 8 ml/kg PBW, MAP decreased and PPV and SVV increased significantly only in responders, resulting in significant differences in MAP, PPV (PPV_8_), and SVV (SVV_8_) between responders and non-responders. Baseline PIP and Crs were comparable between the two groups and increased significantly after V_T_ challenge in responders and non-responders (Table 2).

**Table 2:** Hemodynamic and Respiratory Variables at Baseline, after the Tidal Volume Challenge, before Volume Expansion, and after Volume Expansion in Responders (*n* = 24) and Non-responders (*n* = 14)

	T1 Base 1 (V_T_ 6 ml/kg)	T2 Increased V_T_ (V_T_ 8 ml/kg)	*P1* Value	T3 Base 2 (V_T_ 6 ml/kg)	T4 After VE (V_T_ 6 ml/kg)	*P2* Value
HR (beats/min)						
Responders	74 (68–86)	74 (68–85)	0.492	75 (69–85)	73 (66–82)	0.012
Non-responders	68 (65–77)	68 (65–77)	> 0.99	68 (63–75)	68 (64–77)	> 0.99
MAP (mm Hg)						
Responders	82 ± 10	79 ± 12	0.036	80 ± 13	85 ± 11	0.078
Non-responders	91 ± 13	89 ± 11	> 0.99	87 ± 11	87 ± 10	> 0.99
PIP (cm H_2_O)						
Responders	26 ± 3	31 ± 4	< 0.001	26 ± 3	27 ± 3	< 0.001
Non-responders	25 ± 3	30 ± 4	0.006	25 ± 3	26 ± 3	0.016
Crs (ml/cmH_2_O)						
Responders	17 (14.3–19)	17 (15–19)	0.018	16 (14–19)	15.5 (14–18)	< 0.001
Non-responders	16.5 (13.8–18.3)	17 (14.8–18.3)	0.066	16 (13.8–17.3)	15 (13–17)	0.024
SVI (ml/min^2^)						
Responders	41.5 ± 8.2	40.6 ± 8.9	> 0.99	40.2 ± 9.0	50.6 ± 10.9	< 0.001
Non-responders	50.5 ± 15.2	52.2 ± 15.3	0.864	52 ± 15.7	55.2 ± 16.8	0.018
PPV (%)						
Responders	7 (5.3–8.8)	9 (8–13)*	< 0.001	7.5 (5.3–9.8)*	3.5 (3–5)	< 0.001
Non-responders	5.5 (3.8–6.5)	6 (3.8–7.3)	0.132	5 (3.8–6)	3 (1.8–3.8)	0.006
SVV (%)						
Responders	5 (4–6)	6.5 (4–9)*	< 0.001	6 (4–7)*	4 (3–5)	0.042
Non-responders	5 (2.8–5)	4 (3–5.3)	> 0.99	4 (2.8–5)	4 (2.8–5)	> 0.99

*HR* Heart rate, *RR* Respiratory rate, *MAP* Mean arterial pressure, *PIP* Peak inspiratory pressure, *Crs* Respiratory compliance, *SVI* Stroke volume index, *PPV* Pulse pressure variation, *SVV* Stroke volume variation, *VT Challenge* tidal volume challenge, *VE* Volume expansion

Data are mean ± SD or median (IQR). Patients were considered responders if the stroke volume index increased by at least 15% after volume expansion (6% hydroxyethyl starch 6 ml/kg for 10 min)

*P < 0.05 comparison between responders and non-responders (*n* = 14) at each time point; *P1*-values are for intragroup comparisons of values before (T1) and after the tidal volume challenge (T2); *P2*-values are for intragroup comparisons of values before (T3) and after volume expansion (T4); *P*-values were adjusted using the Bonferroni correction

Significant changes in PIP, Crs, SVI, and PPV were induced in responders and non-responders after VE, while significant decreases in HR and SVV were induced only in responders (Table 2).

### Relationships between changes in PPV and SVV induced by VE and percentage changes in SVI induced by VE

**Fig. 3:**
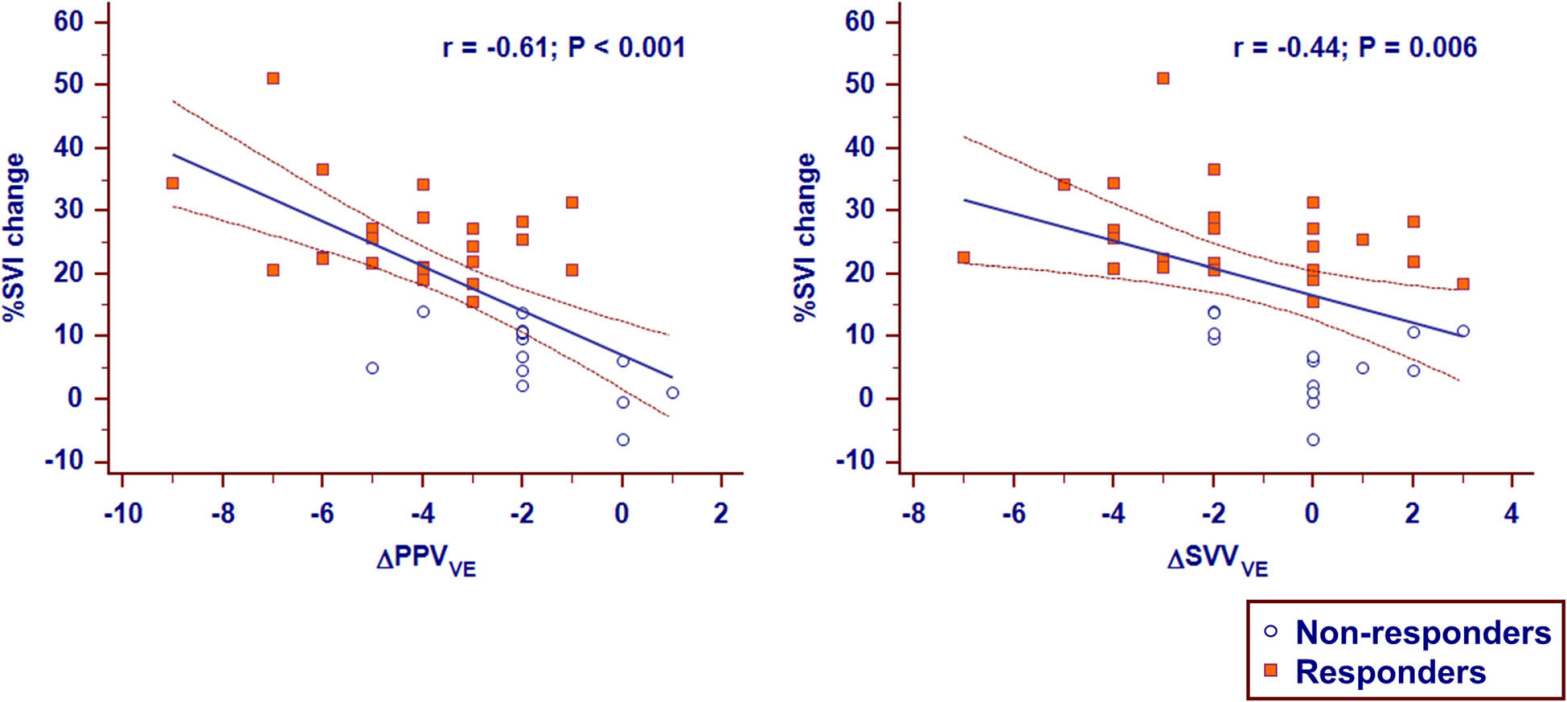
Relationship between volume expansion-induced changes in pulse pressure variation and stroke volume variation and volume expansion-induced percentage changes in the stroke volume index (SVI). ΔPPV_VE_, changes in pulse pressure variation after volume expansion; ΔSVV_VE_, changes in stroke volume variation after volume expansion

PPV_VE_ and ΔSVV_VE_ were significantly correlated with VE-induced percentage changes in SVI (r = − 0.61 [95% CI − 0.78 to − 0.36], *P* < 0.001; r = − 0.44 [95% CI − 0.67 to − 0.14], *P* = 0.006, respectively) (Fig. 3), indicating the ability of these variables to track changes in SVI induced by VE during pneumoperitoneum.

### Prediction of fluid responsiveness

In the ROC curve analysis, ΔPPV_6–8_ showed excellent predictive capability for fluid responsiveness with an AUC of 0.95 (95% CI 0.83–0.99, *P* < 0.0001). ΔPPV_6–8_ > 1% identified responders with sensitivity of 92% (95% CI 73–99) and specificity of 86% (95% CI 57–98). ΔSVV_6–8_ could also predict fluid responsiveness but showed only fair predictive capability, with an AUC of 0.76 (0.60–0.89). The ΔSVV_6–8_ > 2% identified responders with a sensitivity 46% (95% CI 26–67) and specificity of 100% (95% CI 77–100) (Table 3).

**Fig. 4:**
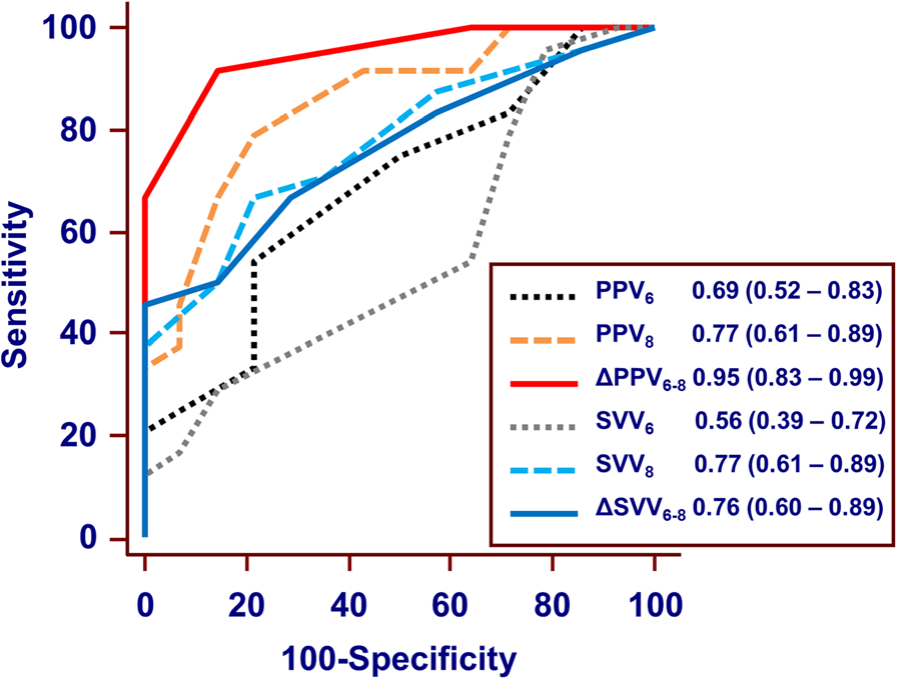
Comparison of receive -operating characteristic curves of PPV_6_, PPV_8_, ΔPPV_6–8,_ SVV_6_, SVV_8_ and ΔSVV_6–8_ to predict fluid responsiveness during robot-assisted laparoscopic surgery in the Trendelenburg position under lung-protective ventilation. PPV_6_, pulse pressure variation during tidal volume at 6 ml/kg predicted body weight (PBW); PPV_8_, pulse pressure variation during tidal volume at 8 ml/kg PBW; ΔPPV_6–8_, change in value of pulse pressure variation after tidal volume challenge; SVV_6_, stroke volume variation during tidal volume at 6 ml/kg predicted body weight (PBW); SVV_8_, stroke volume variation during tidal volume at 8 ml/kg PBW; ΔSVV_6–8_, change in value of stroke volume variation after tidal volume challenge; area under the ROC curve appears in cartouche with 95% confidence interval

PPV_6_ showed poor ability to predict fluid responsiveness, with an AUC of 0.69 (95% CI 0.52–0.83, *P* = 0.036), but PPV_8_ showed good ability, with an AUC of 0.85 (95% CI 0.70–0.95, *P* < 0.0001). SVV_6_ was not predictive of fluid responsiveness, but SVV_8_ showed fair predictive ability, with an AUC of 0.77 (95% CI 0.61–0.89). The optimal threshold values of PPV_8_ and SVV_8_ were 7% [sensitivity 79% (95% CI 58–93); specificity 79% (95% CI 49–95)] and 5% [sensitivity 67% (95% CI 45–84); specificity 79% (95% CI 49–95)], respectively (Table 3). The AUCs for ΔPPV_6–8_ and PPV_8_ were significantly greater than those for PPV_6_ (*P* < 0.001 and *P* = 0.003, respectively). The AUCs for SVV_8_ were significantly greater than those for SVV_6_ (*P* = 0.007) but there were no significant differences in the AUCs for ΔSVV_6–8_ and SVV_6_ (*P* = 0.11). No significant differences were found between the AUCs for ΔPPV_6–8_ and PPV_8_ (*P* = 0.09) or between those for SVV_6–8_ and SVV_8_ (*P* = 0.85) (Fig. 4).

**Table 3:** Prediction of Fluid Responsiveness based on the ROC Curves of Various Indices

	AUC (95%CI)	*P*-value	Cut-off value,%	Sensitivity (95% CI)	Specificity (95% CI)	Grey zone (%)	Patients in the grey zone number (%)
PPV_6_	0.69 (0.52–0.83)	0.036	> 6	54 (33–74)	79 (49–95)	3.2 to 7.8	22 (58)
SVV_6_	0.56 (0.39–0.72)	0.563	–	–	–		
PPV_8_	0.85 (0.70–0.95)	< 0.0001	> 7	79 (58–93)	79 (49–95)	6.2 to 8.6	10 (26)
SVV_8_	0.77 (0.61–0.89)	0.0003	> 5	67 (45–84)	79 (49–95)	2.7 to 6.3	21 (55)
ΔPPV_6–8_	0.95 (0.83–0.99)	< 0.0001	> 1	92 (73–99)	86 (57–98)	1 to 1.3	9 (24)
ΔSVV_6–8_	0.76 (0.60–0.89)	0.0006	> 2	46 (26–67)	100 (77–100)	−1.5 to 1.4	21 (55)
%ΔPPV_6–8_	0.87 (0.72–0.96)	< 0.0001	> 25	83 (63–95)	79 (49–95)	20.5 to 46	12 (32)
%ΔSVV_6–8_	0.71 (0.55–0.85)	0.02	> 16.7	67 (45–85)	79 (49–95)	−32 to 95	27 (71)

*ROC* Receiver operating characteristic, *AUC* Area under the curve, *CI* Confidence interval, *PPV*_*6*_ Pulse pressure variation during tidal volume at 6 ml/kg predicted body weight (PBW), *SVV*_*6*_ Stroke volume variation during tidal volume at 6 ml/kg PBW, *PPV*_*8*_ Pulse pressure variation during tidal volume at 8 ml/kg PBW, *SVV*_*8*_ Stroke volume variation during tidal volume at 8 ml/kg PBW, *ΔPPV*_*6–8*_ Change in value of pulse pressure variation after tidal volume challenge, *ΔSVV*_*6–8*_ Change in value of stroke volume variation after tidal volume challenge, *%ΔPPV*_*6–8*_ Percentage change in value of pulse pressure variation after tidal volume challenge, *%ΔSVV*_*6–8*_ Percentage change in value of stroke volume variation after tidal volume challenge

## Discussion

In this study on patients undergoing robot-assisted laparoscopic surgery in the Trendelenburg position under lung-protective ventilation, we demonstrated that absolute change in PPV values obtained by V_T_ challenge (ΔPPV_6–8_) can predict fluid responsiveness with excellent predictive capability, whereas ΔSVV_6–8_ showed only fair predictive capability. The optimal thresholds of ΔPPV_6–8_ and ΔSVV_6–8_ were > 1% and > 2%, respectively. This study also showed that the augmentation of PPV and SVV following V_T_ challenge improved the predictability of fluid responsiveness compared to PPV and SVV measured with a V_T_ of 6 ml/kg.

Our study demonstrated that a V_T_ challenge improved the predictive power of PPV and SVV values, even in patients with elevated IAP. Because PPV and SVV values are augmented in fluid responders, but not in non-responders, the absolute changes in the PPV and SVV values induced by increased V_T_ could also be used to predict fluid responsiveness, which is consistent with studies of Myatra et al. [19] and Messina et al. [20].

However, the cut-off values of ΔPPV_6–8_ and ΔSVV_6–8_ in our study and in the study of Messina et al. [20] were lower than those reported by Myatra et al. [19]. There are several possible explanations for this discrepancy. The extent of airway pressure transmission to the pleural space is inversely proportional to chest wall compliace (C_cw_) and proportional to lung compliance (C_L_) [33, 34]. Messina et al. described that their cut-off values was lower because Myatra et al. enrolled critically ill patients in the ICU with reduced C_cw_ reported at 30%, while they enrolled healthy surgical patients [20, 35]. As pneumoperitoneum has been known to decreases C_cw_ [36], higher cut-off values could be expected in our study population. However, adding a steep Trendelenburg position to pneumoperitoneum reduces Ccw as well as C_L_ [37], which may have caused the cut off value lower than expected. In addition to the aforementioned factors, different hemodynamic characteristics of the patients may contribute to different cut-off values; Myatra et al. studied critically ill patients with acute circulatory failure, whereas our study population consisted of patients with no clinical signs of shock. That most our study population were in the ‘grey zone’ where responders and non-responders are not well separated [32], would also have contributed to the less profound changes in PPV and SVV values.

Unlike Myatra et al. [19] and Messina et al. [20]., the absolute change in SVV after V_T_ challenge showed only fair ability to predict true fluid responders in our study. This discrepancy may have been attributable to the fact that pneumoperitoneum can induce significant increases in systemic vascular resistance (SVR) [38]. The trending ability of pressure waveform devices to accurately monitor changes in SVI is influenced by SVR [39, 40]. Renner et al. reported that increasing IAP to 25 mmHg abolishes the ability of SVV to predict fluid responsiveness, but not in the case of PPV [12]. This supports our finding that PPV was better able to predict fluid responsiveness than SVV with all of the tested V_T_ values at 6 and 8 ml/kg.

Our study showed discordant results versus those of previous studies regarding the reliability of dynamic preload indices in predicting fluid responsiveness, as well as the appropriate cut-off value [11–15]. These conflicting results can be explained by the fact that the cardiopulmonary interactions that are altered during pneumoperitoneum depend on the level of IAP [41]. Although IAP elevations > 20 mmHg are known to progressively increase the values of dynamic preload indices independent of volume status [12, 13], the lower IAP (10–15 mmHg) in a surgical setting did not modify the cut-off values of dynamic preload indices in the present or previous studies [11, 14, 15]. Unlike the results of Hoiseth et al. [14], which showed relatively poor predictive capability of dynamic preload indices for fluid responsiveness during laparoscopy, we found good predictive capability of PPV at V_T_ 8 ml/kg. This discrepancy can be explained by those researchers not controlling clinical factors, such as blood loss, use of vasopressors, or changes in ventilator settings, which may have altered PPV values independent of the preload condition, whereas we performed VE under controlled clinical conditions with little or no surgical stimulation.

Due to the complex relationship between intrathoracic pressure and IAP, as well as the effect of pressure transmitted to pleura space [42, 43], during pneumoperitoneum, the detection of hypovolemia is particularly difficult. In addition, protective ventilation strategies [18], specifically small V_T_, have been shown to modify the reliability of dynamic preload indices [44]. However, this study showed that the absolute change in PPV after a “V_T_ challenge” reliably predicted fluid responsiveness even under the above limited conditions. As suggested by Myatra et al. [19], the V_T_ challenge can be applied in resource-limited settings as this test does not require a continuous cardiac output monitor. Because ΔPPV_VE_ were significantly correlated with percentage changes in SVI induced by VE (Fig. 3), fluid responsiveness after VE also can be confirmed without continuous cardiac output monitoring.

Nevertheless, 24% of patients were included in the grey zone analysis of ΔPPV_6–8_, suggesting caution in using of the single cutoff (ΔPPV_6–8_ > 1%) as the sole target in hemodynamic management. Furthermore, hemodynamic resuscitation should be aimed at achieving not only adequate SV but also sufficient MAP for the body to maintain tissue oxygenation [45]. Because the arterial pressure response VE depends on arterial tone, knowing whether a patient is preload-dependent provides only a partial solution to the problem [46]. In this regard, functional assessment of arterial load by dynamic arterial elastance (Eadyn), defined as the ratio between PPV and SVV has recently been shown to predict the arterial pressure response to VE in hypotensive, preload-dependent patients [47]. Further studies are needed to determine whether this parameter is effective in pneumoperitoneum, using time-synced data from patient monitor and cardiac output monitor [48].

This study had several limitations. First, the study population consisted of only a small number of highly selected patients receiving robot-assisted laparoscopic surgery in the Trendelenburg position. As the IAP was maintained at 15 mmHg, our results cannot be extrapolated to different IAP values. Our results require validation in a larger and more heterogeneous population. Second, we performed the V_T_ challenge for 3 min, not 1 min as described previously [19]. We used an extended time because we have assumed that extending the time for V_T_ challenge may have sensitized the test and possibly led to more observations of increases in PPV and SVV. However, during extended time, some equilibrium or compensatory effects could be occurred, which may be an important reason to explain the difference in our cut off values from the study of Myatra et al. [19]. Third, there were sex differences between the responders and non-responders, which may have been due to differences in types of surgery based on sex, resulting in different hemodynamic status at the start of the study protocol. However, we considered it unlikely that this difference would have affected our primary outcome in predicting the fluid response during robot-assisted laparoscopic surgery in the Trendelenburg position because the IAP and angle of Trendelenburg position during surgery were constant in all patients.

## Conclusions

In conclusion, the change in PPV following the V_T_ challenge test has excellent reliability in predicting fluid responsiveness in patients undergoing robot-assisted laparoscopic surgery in the Trendelenburg position under lung-protective ventilation. The change in SVV and absolute values of PPV and SVV following this test are also reliable.

## Data Availability

The datasets generated and/or analysed during the current study are not publicly available due to the regulation of Institutional Review Board, but are available from the corresponding author after getting permission from IRB for sharing the dataset on reasonable request.

## Abbreviations

AUC: Area under the curve
BMI: Body mass index
CI: Confidence interval
Crs: Compliance of the respiratory system
ECG: Electrocardiography
HR: Heart rate
IAP: Intra-abdominal pressure
MAP: Mean arterial pressure
ODM: Oesophageal Doppler monitor
PBW: Predicted body weight
PEEP: Positive end expiratory pressure
PIP: Peak inspiratory airway pressure Pulse pressure variation
PP: Pulse pressure
PV: Peak velocity
RBF: Renal blood flow
SVI: Stroke volume index
SVV: Stroke volume variation
VE: Volume expansion
V_T_: Tidal volume
